# Supplementary material for: Longitudinal plasma neurofilament light chain and patient-reported outcomes as complementary markers of vincristine-associated peripheral neuropathy in adults with lymphoma: a cohort study
Source: medRxiv. 2026 Jul 1:2026.06.28.26356741. Preprint. [Version 1] doi: 10.64898/2026.06.28.26356741 (PMC13345425; doi:10.64898/2026.06.28.26356741)
Supplement: Supplementary file 1 [file NIHPP2026.06.28.26356741V1-supplement-1.pdf]

medRxiv preprint doi: https://doi.org/10.64898/2026.06.28.26356741; this version posted July 1, 2026. The copyright holder for this preprint (which was not certified by peer review) is the author/funder, who has granted medRxiv a license to display the preprint in perpetuity. It is made available under a CC-BY 4.0 International license .

Supplemental figure and table legends

Figure S1. Primary NfL model displayed on the log10 scale.

Model-estimated plasma NfL and 95% confidence intervals from the primary mixed-effects model shown directly on the log10 scale. The model included timepoint as a fixed effect and a participant-level random intercept. Points show model estimates and the shaded band shows the 95% confidence interval. P values are model-based estimated marginal mean contrast p values comparing each follow-up timepoint with T1/Pre-C1. Compared with T1/Pre-C1, log10 NfL was higher at T2/Pre-C2 (p = 4.16e-06) T3/Pre-C4 (p = 6.41e-10) and T4/Pre-C6 (p = 3.39e-08).

Figure S2. Plasma NfL trajectories by chemotherapy regimen.

Model-estimated plasma NfL geometric means and 95% confidence intervals by chemotherapy regimen, back-transformed to pg/mL from an exploratory mixed-effects model including timepoint, regimen, and timepoint-by-regimen interaction terms. Points show back-transformed model estimates, and vertical error bars show 95% confidence intervals.

Statistical testing used model-based fixed-effect and interaction terms from the exploratory regimen model. The main effect for EPOCH compared with CHOP was not significant (p = 0.779). Timepoint-by-regimen interaction terms were not significant at T2/Pre-C2 (p = 0.132) T3/Pre-C4 (p = 0.929) or T4/Pre-C6 (p = 0.356) compared with T1/Pre-C1. CHOP, cyclophosphamide, doxorubicin, vincristine, and prednisone; EPOCH, etoposide, prednisone, vincristine, cyclophosphamide, and doxorubicin; NfL, neurofilament light chain.

Figure S3. Exploratory vincristine dose-modification analyses.

(A) Change in log10 plasma NfL from baseline to T4/Pre-C6 stratified by whether participants had any vincristine dose modification by T4/Pre-C6. (B) Change in raw CIPN20 total score from baseline to T4/Pre-C6 stratified by any vincristine dose modification (including discontinuation) by T4/Pre-C6.

Boxes show the interquartile range, horizontal lines show the median, whiskers show the observed minimum and maximum, and points show individual participants. Mann-Whitney U tests.

(A) all-participant log10 NfL analysis included n = 11 (3 dose-modified, 8 with no modification) p = 0.1939. (B) all-participant CIPN20 analysis included n = 14 (5 dose-modified, 9 with no modification) p = 0.1381. The high CIPN20-change outlier was in the no-modification group with delta CIPN20 = +35 from baseline to T4/Pre-C6; this was an IQR-rule outlier within the no-modification group but was retained because it may associate with an observed clinical value. NfL, neurofilament light chain; CIPN20, Chemotherapy-Induced Peripheral Neuropathy questionnaire.ΔΔ

Table S1. Timepoint-specific associations between plasma NfL and CIPN20 scores

Spearman correlations between paired log10 plasma NfL and CIPN20 total score are shown separately forT1/Pre-C1,T2/Pre-C2,T3/Pre-C4, andT4/Pre-C6. These analyses were exploratory and underpowered, and were used to contextualize the pooled visit-level association shown in Figure 5A. NfL, neurofilament light chain; CIPN20, Chemotherapy-Induced Peripheral Neuropathy questionnaire.

medRxiv preprint doi: https://doi.org/10.64898/2026.06.28.26356741; this version posted July 1, 2026. The copyright holder for this preprint (which was not certified by peer review) is the author/funder, who has granted medRxiv a license to display the preprint in perpetuity.
It is made available under a CC-BY 4.0 International license .

Table S2. Subject-level vincristine dose-modification and treatment-period clinical context

Rows show subject-level visit data for vincristine dose modification, CIPN20 total and sensory scores, BPI-SF, CTCAE, NfL, and the added baseline cancer stage and HbA1c/eA1c context. HbA1c/eA1c combines measured HbA1c with glucose-derived estimated HbA1c when HbA1c was unavailable; glucose-derived eA1c values were used descriptively only. ND: not documented; CIPN20, Chemotherapy-Induced Peripheral Neuropathy 20-item questionnaire; BPI-SF, Brief Pain Inventory-Short Form; CTCAE, Common Terminology Criteria for Adverse Events; NfL, neurofilament light chain; HbA1c, hemoglobin A1c; eA1c, estimated HbA1c.

Table S3. Exploratory T5/Post patient-reported outcomes and dose-modification context

Long-term patient-reported outcomes are shown for participants with available T5/Post data 24-42 months after completion of vincristine-containing chemotherapy. Treatment-period CIPN20, T5/Post CIPN20, T5/Post BPI-SF, T3 CTCAE grade, available treatment-period plasma NfL values, and vincristine dose-modification history are presented descriptively. NfL, neurofilament light chain; CIPN20, Chemotherapy-Induced Peripheral Neuropathy questionnaire; CTCAE, Common Terminology Criteria for Adverse Events; BPI-SF, Brief Pain Inventory-Short Form.

medRxiv preprint doi: https://doi.org/10.64898/2026.06.28.26356741; this version posted July 1, 2026. The copyright holder for this preprint (which was not certified by peer review) is the author/funder, who has granted medRxiv a license to display the preprint in perpetuity.
It is made available under a CC-BY 4.0 International license .

951 **Figure S1. Primary NfL model displayed on the log10 scale**

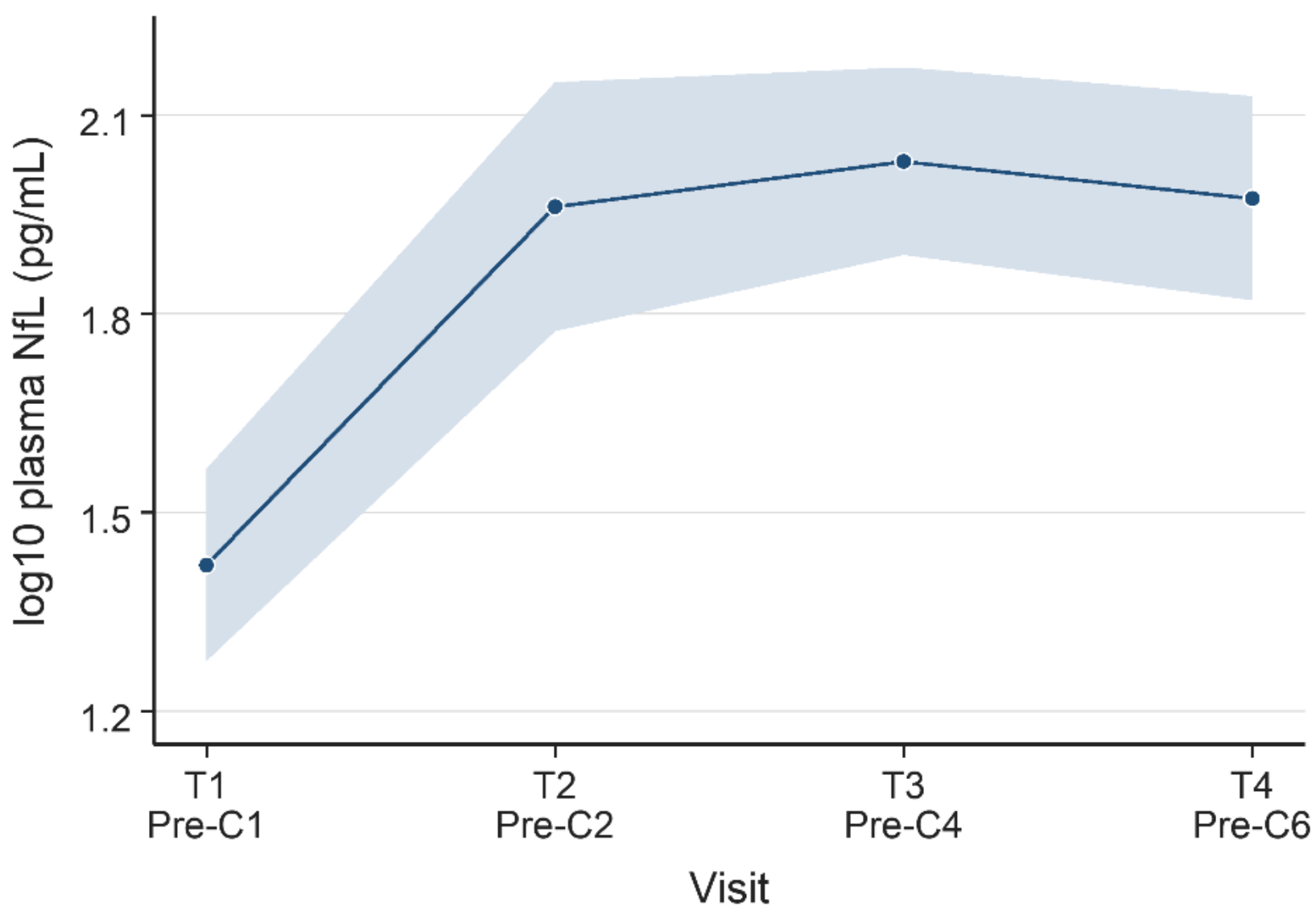

medRxiv preprint doi: https://doi.org/10.64898/2026.06.28.26356741; this version posted July 1, 2026. The copyright holder for this preprint
(which was not certified by peer review) is the author/funder, who has granted medRxiv a license to display the preprint in perpetuity.
It is made available under a CC-BY 4.0 International license .

Figure S2. Plasma NfL trajectories by chemotherapy regimen

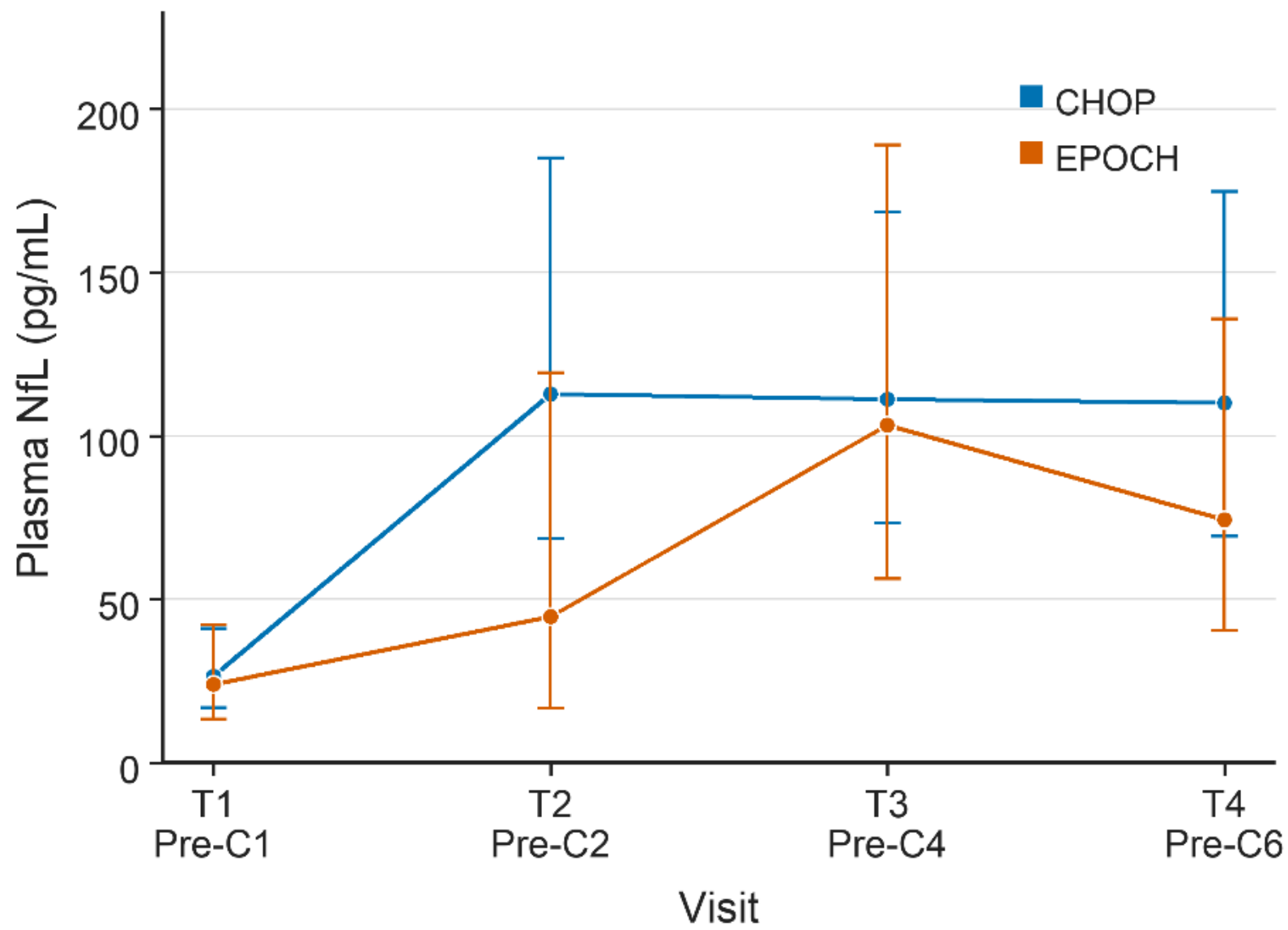

medRxiv preprint doi: https://doi.org/10.64898/2026.06.28.26356741; this version posted July 1, 2026. The copyright holder for this preprint (which was not certified by peer review) is the author/funder, who has granted medRxiv a license to display the preprint in perpetuity.
It is made available under a CC-BY 4.0 International license .

Figure S3. Exploratory vincristine dose-modification analyses.

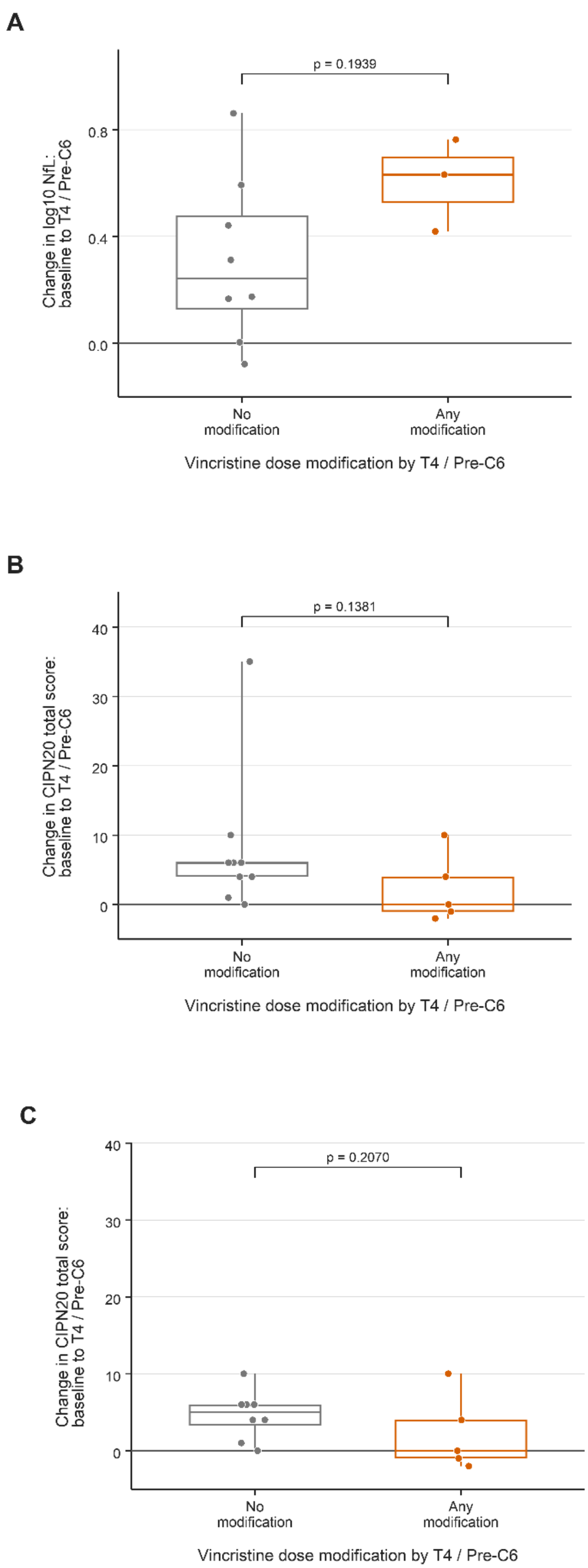

medRxiv preprint doi: https://doi.org/10.64898/2026.06.28.26356741; this version posted July 1, 2026. The copyright holder for this preprint (which was not certified by peer review) is the author/funder, who has granted medRxiv a license to display the preprint in perpetuity.
It is made available under a CC-BY 4.0 International license .

Table S1. Timepoint-specific association between log10 NfL and CIPN20 scores

Timepoint	N	Spearman ρ	p value
T1/Pre-C1	16	-0.358	0.174
T2/Pre-C2	7	0.891	0.007
T3/Pre-C4	17	0.399	0.112
T4/Pre-C6	11	-0.119	0.728

Table S2. Subject-level vincristine dose-modification, CIPN20 sensory, and treatment-period biomarker context

Code	Treat-ment	Stage	HbA1c/eA1c (%)	HbA1c/eA1c source	HbA1c/eA1c category	Timepoint	Vincristine dose modification	Dose-modification reason	CIPN 20 total	CIPN 20 sensory	BPI-SF	CTCAE	NfL (pg/mL)
P01	CHOP	IV	5.6	Measured HbA1c	Normal	T1/Pre-C1	No modification	Not applicable	19	9	1.5	0	69.9
P02	CHOP	IV	6.9	Glucose-derived eA1c	Diabetes range	T1/Pre-C1	No modification	Not applicable	21	10	1	0	187.9
P03	CHOP	IV	5.1	Measured HbA1c	Normal	T2/Pre-C2	No modification	Not applicable	25	10	2.5	0	101.6
P03	CHOP	IV	5.1	Measured HbA1c	Normal	T3/Pre-C4	No modification	Not applicable	25	11	4.75	0	96.3
P03	CHOP	IV	5.1	Measured HbA1c	Normal	T4/Pre-C6	No modification	Not applicable	31	14	5.25	0	Missing
P04	EPOCH	IV	5.4	Measured HbA1c	Normal	T1/Pre-C1	No modification	Not applicable	25	10	0	1	12.9
P04	EPOCH	IV	5.4	Measured HbA1c	Normal	T3/Pre-C4	Removed	CIPN	32	14	0	2	175.5
P04	EPOCH	IV	5.4	Measured HbA1c	Normal	T4/Pre-C6	Removed	CIPN	35	13	0	1	75
P05	CHOP	IV	7.0	Measured HbA1c	Diabetes range	T1/Pre-C1	No modification	Not applicable	19	9	0	0	17.9
P05	CHOP	IV	7.0	Measured HbA1c	Diabetes range	T3/Pre-C4	No modification	Not applicable	27	9	1.75	0	70.3
P06	EPOCH	II	4.7	Glucose-derived eA1c	Normal	T1/Pre-C1	No modification	Not applicable	21	9	0	0	63.1
P06	EPOCH	II	4.7	Glucose-derived eA1c	Normal	T3/Pre-C4	No modification	Not applicable	19	9	0	0	Missing
P06	EPOCH	II	4.7	Glucose-derived eA1c	Normal	T4/Pre-C6	No modification	Not applicable	25	10	0	0	94.4
P07	CHOP	III	6.0	Measured HbA1c	Prediabetes	T1/Pre-C1	No modification	Not applicable	19	9	0	0	23.7
P07	CHOP	III	6.0	Measured HbA1c	Prediabetes	T3/Pre-C4	No modification	Not applicable	20	9	2	0	114.3
P07	CHOP	III	6.0	Measured HbA1c	Prediabetes	T4/Pre-C6	No modification	Not applicable	25	10	0.75	1	172.1
P08	EPOCH	ND	5.4	Measured HbA1c	Normal	T1/Pre-C1	No modification	Not applicable	25	10	Missing	0	Missing
P09	EPOCH	ND	4.2	Measured HbA1c	Normal	T1/Pre-C1	No modification	Not applicable	20	10	0.25	0	72.4
P09	EPOCH	ND	4.2	Measured HbA1c	Normal	T3/Pre-C4	No modification	Not applicable	21	10	0.5	1	Missing
P09	EPOCH	ND	4.2	Measured HbA1c	Normal	T4/Pre-C6	50% reduction	constipation	20	10	0.25	1	189.9
P10	CHOP	IV	7.3	Measured HbA1c	Diabetes range	T2/Pre-C2	No modification	Not applicable	21	10	0	0	80.1

medRxiv preprint doi: https://doi.org/10.64898/2026.06.28.26356741; this version posted July 1, 2026. The copyright holder for this preprint (which was not certified by peer review) is the author/funder, who has granted medRxiv a license to display the preprint in perpetuity.
It is made available under a CC-BY 4.0 International license .

Code	Treat-ment	Stage	HbA1c/eA1c (%)	HbA1c/eA1c source	HbA1c/eA1c category	Timepoint	Vincristine dose modification	Dose-modification reason	CIPN 20 total	CIPN 20 sensory	BPI-SF	CTCAE	NfL (pg/mL)
P10	CHOP	IV	7.3	Measured HbA1c	Diabetes range	T3/Pre-C4	No modification	Not applicable	25	11	0	0	80
P10	CHOP	IV	7.3	Measured HbA1c	Diabetes range	T4/Pre-C6	No modification	Not applicable	27	12	0	0	80.8
P11	CHOP	IV	6.8	Measured HbA1c	Diabetes range	T1/Pre-C1	No modification	Not applicable	20	9	0	Missing	31.4
P11	CHOP	IV	6.8	Measured HbA1c	Diabetes range	T3/Pre-C4	No modification	Not applicable	21	9	0	0	232.2
P12	EPOCH	ND	6.0	Measured HbA1c	Prediabetes	T1/Pre-C1	No modification	Not applicable	22	11	0	0	58.9
P13	CHOP	ND	6.5	Glucose-derived eA1c	Diabetes range	T1/Pre-C1	No modification	Not applicable	21	9	2.5	0	16.1
P13	CHOP	ND	6.5	Glucose-derived eA1c	Diabetes range	T3/Pre-C4	No modification	Not applicable	26	15	0	1	99.9
P13	CHOP	ND	6.5	Glucose-derived eA1c	Diabetes range	T4/Pre-C6	No modification	Not applicable	31	14	0	1	63.2
P14	CHOP	IV	6.0	Measured HbA1c	Prediabetes	T2/Pre-C2	25% reduction	CIPN	21	10	0.5	2	74.3
P14	CHOP	IV	6.0	Measured HbA1c	Prediabetes	T3/Pre-C4	25% reduction	CIPN	23	11	4	1	Missing
P14	CHOP	IV	6.0	Measured HbA1c	Prediabetes	T4/Pre-C6	50% reduction	CIPN	25	12	4.5	2	Missing
P15	CHOP	IV	4.7	Glucose-derived eA1c	Normal	T2/Pre-C2	No modification	Not applicable	19	9	0	0	68.7
P15	CHOP	IV	4.7	Glucose-derived eA1c	Normal	T3/Pre-C4	No modification	Not applicable	18	9	0	0	62.6
P15	CHOP	IV	4.7	Glucose-derived eA1c	Normal	T4/Pre-C6	No modification	Not applicable	19	9	0	0	57.4
P16	CHOP	IIIA	6.2	Measured HbA1c	Prediabetes	T1/Pre-C1	No modification	Not applicable	20	9	0	Missing	19.7
P16	CHOP	IIIA	6.2	Measured HbA1c	Prediabetes	T3/Pre-C4	50% reduction	Multifactorial	24	9	1.75	1	74.8
P16	CHOP	IIIA	6.2	Measured HbA1c	Prediabetes	T4/Pre-C6	50% reduction	Multifactorial	19	9	4	0	84.3
P17	EPOCH	IV	4.3	Measured HbA1c	Normal	T1/Pre-C1	No modification	Not applicable	20	9	0	0	19.2
P17	EPOCH	IV	4.3	Measured HbA1c	Normal	T3/Pre-C4	No modification	Not applicable	20	9	0	0	58
P17	EPOCH	IV	4.3	Measured HbA1c	Normal	T4/Pre-C6	No modification	Not applicable	21	9	0	0	53.1
P18	EPOCH	IV	4.5	Measured HbA1c	Normal	T2/Pre-C2	No modification	Not applicable	19	9	0	0	36.2
P18	EPOCH	IV	4.5	Measured HbA1c	Normal	T3/Pre-C4	No modification	Not applicable	35	20	7.75	1	63.9
P18	EPOCH	IV	4.5	Measured HbA1c	Normal	T4/Pre-C6	No modification	Not applicable	54	24	4	0	74.4
P19	CHOP	IV	6.3	Glucose-derived eA1c	Prediabetes	T2/Pre-C2	No modification	Not applicable	20	9	Missing	0	57.7
P19	CHOP	IV	6.3	Glucose-derived eA1c	Prediabetes	T3/Pre-C4	No modification	Not applicable	21	9	0	0	Missing
P19	CHOP	IV	6.3	Glucose-derived eA1c	Prediabetes	T4/Pre-C6	No modification	Not applicable	24	9	0	0	84.6
P20	EPOCH	ND	5.5	Glucose-derived eA1c	Normal	T1/Pre-C1	No modification	Not applicable	22	9	0	0	12.1
P20	EPOCH	ND	5.5	Glucose-derived eA1c	Normal	T3/Pre-C4	Removed	Ileus	18	9	0	0	32.1
P20	EPOCH	ND	5.5	Glucose-derived eA1c	Normal	T4/Pre-C6	Removed	Ileus	20	9	0	0	Missing

medRxiv preprint doi: https://doi.org/10.64898/2026.06.28.26356741; this version posted July 1, 2026. The copyright holder for this preprint (which was not certified by peer review) is the author/funder, who has granted medRxiv a license to display the preprint in perpetuity.
It is made available under a CC-BY 4.0 International license .

Code	Treat-ment	Stage	HbA1c/eA1c (%)	HbA1c/eA1c source	HbA1c/eA1c category	Timepoint	Vincristine dose modification	Dose-modification reason	CIPN 20 total	CIPN 20 sensory	BPI-SF	CTCAE	NfL (pg/mL)
P21	CHOP	IV	8.5	Measured HbA1c	Diabetes range	T2/Pre-C2	No modification	Not applicable	22	9	0	0	142.2
P21	CHOP	IV	8.5	Measured HbA1c	Diabetes range	T3/Pre-C4	No modification	Not applicable	21	9	0	1	67.4
P22	CHOP	ND	4.7	Measured HbA1c	Normal	T2/Pre-C2	No modification	Not applicable	22	11	0	0	Missing
P22	CHOP	ND	4.7	Measured HbA1c	Normal	T3/Pre-C4	No modification	Not applicable	20	9	0	0	37
P22	CHOP	ND	4.7	Measured HbA1c	Normal	T4/Pre-C6	No modification	Not applicable	Missing	Missing	Missing	0	34
P23	EPOCH	IIA	5.4	Measured HbA1c	Normal	T1/Pre-C1	No modification	Not applicable	21	9	0	0	6.8
P23	EPOCH	IIA	5.4	Measured HbA1c	Normal	T3/Pre-C4	No modification	Not applicable	26	13	0	0	65.7
P24	CHOP	III-IV	5.4	Measured HbA1c	Normal	T1/Pre-C1	25% reduction	Not specified	18	9	Missing	0	71.5
P24	CHOP	III-IV	5.4	Measured HbA1c	Normal	T3/Pre-C4	25% reduction	Not specified	26	10	2.25	0	212.8
P25	CHOP	ND	5.0	Measured HbA1c	Normal	T1/Pre-C1	No modification	Not applicable	21	9	3.25	0	25.9
P25	CHOP	ND	5.0	Measured HbA1c	Normal	T3/Pre-C4	No modification	Not applicable	25	9	5	0	208.6

Table S3. Exploratory Post/T5 patient-reported outcomes and vincristine dose-modification context

Code	Treat-ment	Months since comple-tion	T4/Pre-C6 CIPN20 total	T5/Post CIPN20 total	T5/Post CIPN20 sensory	T5/Post BPI-SF	T4/Pre-C6 CTCAE	Dose modifica-tion	% reduction/ removal	Timing/ reason	NfL T1/ Pre-C1	NfL T2/ Pre-C2	NfL T3/ Pre-C4	NfL T4/ Pre-C6
P06	EPOCH	28	25	20	9	0.25	0	No	No modification		63.1			94.4
P09	EPOCH	42	20	28	16	0	1	Yes	50% reduction	C6; constipation	72.4			189.9
P13	CHOP	38	31	25	13	3.5	1	No	No modification		16.1		99.9	63.2
P14	CHOP	24	25	36	21	4.25	2	Yes	25% reduction, 50% reduction	C2/C5; CIPN		74.3		
P16	CHOP	39	19	21	10	1.5	0	Yes	50% reduction	C4; multifactorial	19.7		74.8	84.3
P18	EPOCH	38	54	22	9	2.5	0	No	No modification			36.2	63.9	74.4
